# Roles unveiled for membrane-associated mucins at the ocular surface using a Muc4 knockout mouse model

**DOI:** 10.1038/s41598-023-40491-0

**Published:** 2023-08-21

**Authors:** Rafael Martinez-Carrasco, Satyanarayan Rachagani, Surinder K. Batra, Pablo Argüeso, M. Elizabeth Fini

**Affiliations:** 1 New England Eye Center, Tufts Medical Center and Department of Ophthalmology, Tufts University School of Medicine, Boston, MA 02111 USA; 2 Department of Biochemistry & Molecular Biology, University of Nebraska Medical Center, Omaha, NE USA; 3 Department of Pathology, University of Nebraska Medical Center, Omaha, NE USA; 4 Buffett Cancer Center, Eppley Institute for Research in Cancer and Allied Diseases, University of Nebraska Medical Center, Omaha, NE USA; 5 Program in Immunology, Tufts Graduate School of Biomedical Sciences, Tufts University, Boston, MA USA; 6 Program in Genetics, Molecular & Cellular Biology, Tufts Graduate School of Biomedical Sciences, Tufts University, Boston, MA USA; 7 Program in Pharmacology & Drug Development, Tufts Graduate School of Biomedical Sciences, Tufts University, Boston, MA USA

**Keywords:** Biochemistry, Molecular biology, Physiology

## Abstract

Membrane-associated mucins (MAMs) are proposed to play critical roles at the ocular surface; however, in vivo evidence has been lacking. Here we investigate these roles by phenotyping of a Muc4 KO mouse. Histochemical analysis for expression of the beta-galactosidase transgene replacing Muc4 revealed a spiraling ribbon pattern across the corneal epithelium, consistent with centripetal cell migration from the limbus. Depletion of Muc4 compromised transcellular barrier function, as evidenced by an increase in rose bengal staining. In addition, the corneal surface was less smooth, consistent with disruption of tear film stability. While surface cells presented with well-developed microprojections, an increase in the number of cells with fewer microprojections was observed. Moreover, an increase in skin-type keratin K10 and a decrease in transcription factor Pax6 was observed, suggesting an incipient transdifferentiation. Despite this, no evidence of inflammatory dry eye disease was apparent. In addition, Muc4 had no effect on signaling by toll-like receptor Tlr4, unlike reports for MUC1 and MUC16. Results of this study provide the first in vivo evidence for the role of MAMs in transcellular barrier function, tear film stability, apical epithelial cell architecture, and epithelial mucosal differentiation at the ocular surface.

## Introduction

The wet ocular surface comprises the corneal and conjunctival epithelia, and their adnexa, as well as the overlying tear film that maintains their wetness^1^. Wet epithelial surfaces throughout the body are protected by a layer of mucus^2^. This complex biological substance is critical for maintaining tissue hydration. The physicochemical properties of mucus are mainly determined by the presence of mucins, large glycoproteins that contain numerous segments of serine and threonine-rich tandem repeats of amino acids. These residues serve as sites for *O*-glycosylation; the resulting long, branched *O*-glycan chains provide mucins with water-holding properties^3–5^.

Mucins can be secreted or membrane-associated. The secreted mucins, which are produced by specialized goblet cells, can assemble into extremely large oligomeric gels via disulfide bonds^3^. In this form, they create a viscous mucus layer over the epithelia of the tracheobronchial, gastrointestinal, and reproductive tracts. However, at the ocular surface, they assemble into a muco-aqueous gel which imparts transparency and fluidity to the tear film^6,7^. This watery gel is surfaced by a layer of lipid, protecting against evaporation^8^. Membrane-associated mucins (MAMs) integrate into the plasma membrane and project their extracellular domains out from the apical surface of corneal and conjunctival epithelia. In this way, they form that major component of the glycocalyx that comprises the deepest tear film compartment^6,9^. In addition, their ectodomains (EDs) can be shed into the muco-aqueous gel of the tear film by specific cleavage near the transmembrane domain^5,10^.

Evidence from biophysical modeling and cell culture studies suggests that both secreted mucins and MAMs contribute to tear film stability and spreading by providing shear thinning properties to tears, reducing friction during blinks, and enhancing corneal wettability wettability^7,11,12^. Instability of the tear film results in dry eye, a common affliction that affects 5% to 34% of people globally^13^. Numerous observational studies have reported that secreted mucins, and MAMs are quantitatively or qualitatively deficient in this disease; however, their contribution to dry eye pathology remains poorly defined^14–16^.

The heavily glycosylated EDs of some MAMs are exceptionally long^17–19^. The longest in humans is MUC16 at 14,517 amino acids in length; the second longest is MUC4 at 7418 amino acids in length. In contrast, MUC1 is only 481 amino acids in length^17,18^. MUC4 and MUC16 are the MAMs with very long EDs expressed at the ocular surface^17,18^. Because of the large number of *O*-glycans on MAMs with very long EDs, they have been hypothesized to play a role in transcellular barrier function. Indeed, MUC16 knockdown in a cell culture model demonstrated a decrease in transcellular barrier function^20^, while knockdown of MUC1, a short ED MAM, did not^21^. Likewise, the knockdown of MUC16, but not MUC1, disrupted the actin cytoskeleton associated with tight junctions and reduced plasma membrane microprojections^21^. These findings suggest that MAMs with very long EDs have specialized roles that MAMs do not serve with short EDs^22^.

While MAM properties conferred by the *O*-glycan chains have received much attention, it is increasingly appreciated that MAMs also serve as cell surface receptors that sense the extracellular environment and transduce signals intracellularly. The binding of signaling proteins and phosphorylation occurs at sites in both the EDs and the cytoplasmic tails (CTs)^17,18,23,24^. Toll-like receptors sense danger signals and pathogen-associated molecular patterns intrinsic to microorganisms and initiate an innate immune response^25^. MUC1 was shown to dampen the inflammatory response after TLR5 activation by blocking its binding to MyD88^26^. This finding was confirmed and extended in a human corneal epithelial cell culture model, where it was found that knockdown of either MUC1 or MUC16 dampened expression of the proinflammatory cytokines TNFA, IL6 and IL8 in response to ligand-activated TLR5^27^.

Transgenic knockout (KO) mouse lines have provided useful models for ocular surface disease^28–30^ and have made it possible to evaluate roles for specific genes (e.g.,^31^). There are currently three published studies on the ocular surface phenotype of MAM KO mice, two of which utilized the *Muc1* KO mouse. Increased susceptibility to infection was noted in the first study^32^; however, the second study (which used a different genetic background) found no evidence of this or any other phenotype^33^. This was not due to the masking of the phenotype by compensatory upregulation of other mucin genes^33^.

The third study examined the phenotype of the *Muc16* KO mouse^34^. Upregulation of inflammatory signaling and features of an ongoing repair process was observed in the ocular surface epithelia of KO mice; however, staining with the clinical dye fluorescein, which is used to measure superficial punctate keratopathy in dry eye, was unchanged. Rose bengal exclusion was not evaluated. No change in the architecture of cell surface microplicae was observed^34^.

Like humans, mice express both *Muc4* and *Muc16* at the ocular surface but with somewhat different localization patterns. Muc4 appears to substitute for Muc16 in the mouse corneal epithelium, suggesting that the *Muc4* KO mouse might be more revealing of roles proposed for MAMs with very long EDs. In the present study, we investigated the role of Muc4 at the ocular surface using a *Muc4* KO mouse recently created in one of our labs^35^.

## Results

### Histochemistry, gross analysis and histology

**Figure 1:**
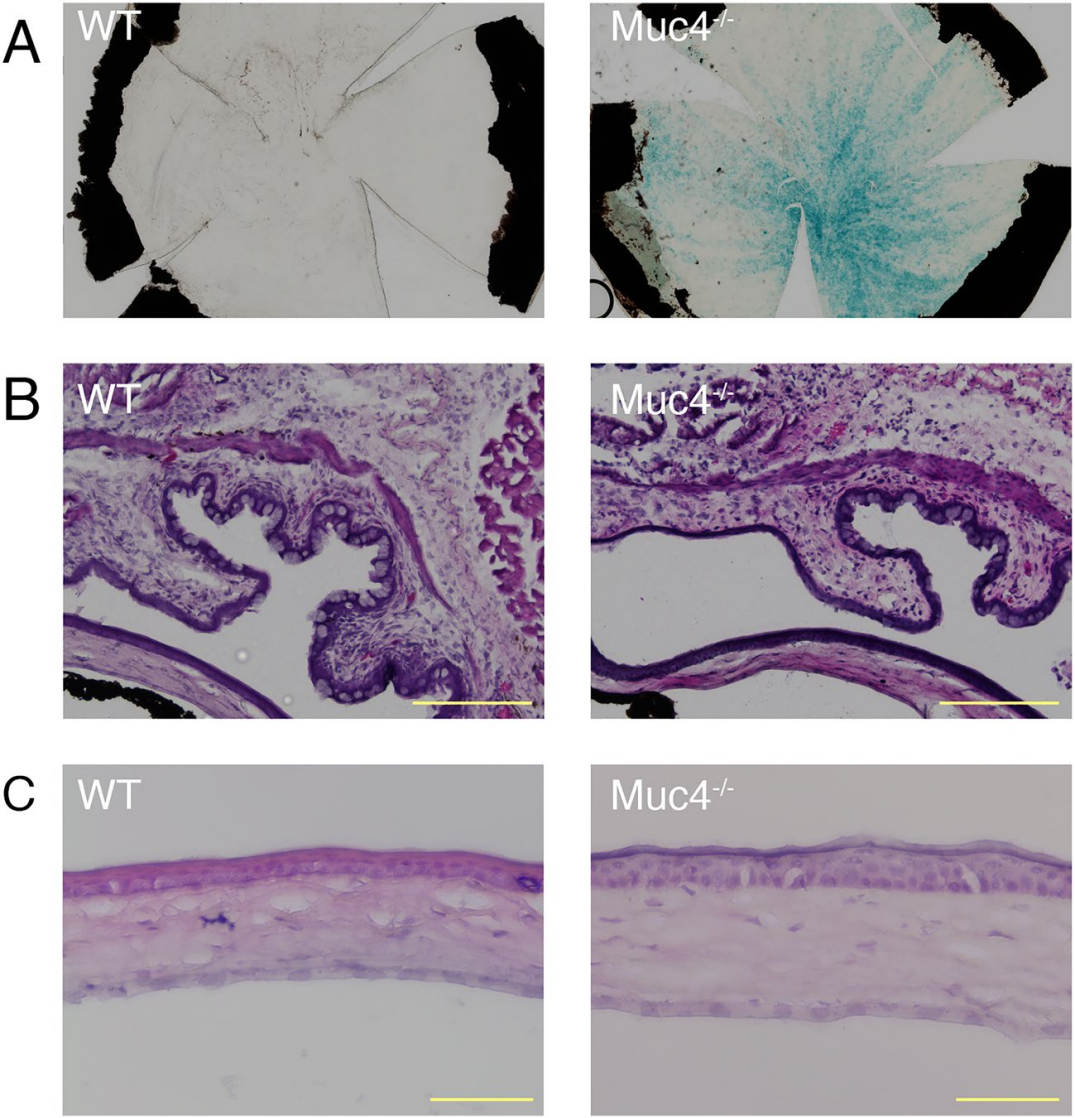
Histologic analysis of *Muc4* KO corneas. (**A**) X-gal staining on whole mount corneas showing activation of the *Muc4* promoter along the corneal surface. (**B**, **C**) Representative H&E-stained cross-sectional images from WT and Muc4 KO mouse eyes showing (**B**) conjunctiva; scale bar = 200 µM and (**C**) cornea; scale bar = 100 µM; N = 5.

We began our investigation of an ocular surface phenotype for the *Muc4* KO mouse using histochemical, gross analysis, and histological methods. Representative results are shown in Fig. 1.

The *Muc4* targeting approach for the mice used in this study utilized a knock-in strategy, inserting a bacterial beta-galactosidase (LacZ) transgene in the endogenous *Muc4* locus, placing it under the control of the *Muc4* promoter. Histochemical analysis for LacZ activity can then be used to confirm the disruption of the endogenous *Muc4* gene in cells where *Muc4* would normally be expressed. In the original study describing these mice, the expected beta-galactosidase activity was observed in the colon and testes, where *Muc4* is expressed, while no activity was observed in the pancreas, where *Muc4* is not expressed^35^. We performed a similar histochemical analysis of the corneal surface, comparing *Muc4*^−/−^ and WT littermate mice (Fig. 1A). Blue staining indicating beta-galactosidase activity was clearly present in epithelial cells at the corneal surface of *Muc4*^−/−^ mice, but absent in WT mice. The beta-galactosidase activity was observed throughout the corneal epithelium, consistent with previous qPCR expression studies^33,36^. However, a new finding was made possible because the 2D coronal view showed that expression was not uniform across the ocular surface. A spiraling pattern was observed, consistent with the known centripetal migration of epithelial cells from the limbus at the corneal periphery to the central cornea^37^. Staining was ribbon-like, with darker and lighter areas, suggesting that the level of *Muc4* expression beginning around the circumference of the limbus must vary. Staining presented with increasing intensity from the periphery towards the central cornea, which was not previously reported.

The previous study from one of our labs describing development of the *Muc4*^−/−^ mice found that they are viable and fertile with no obvious anatomical defects^35^. In an examination of the ocular surface of the *Muc4*^−/−^ mouse eye via stereomicroscopy, we also found no apparent abnormalities of the corneal epithelium, conjunctival epithelia or eyelids, and no evidence of conjunctivitis or blepharitis. Hematoxylin and eosin staining of cross-sections through conjunctiva (Fig. 1B) and cornea (Fig. 1C) of *Muc4*^−/−^ and WT mice also revealed no evidence of inflammation, and there were no apparent morphological differences. The conjunctival epithelium of *Muc4*^−/−^ mice appeared normal and goblet cell density was similar in both WT and *Muc4*^−/−^ mice (Fig. 1B). The corneal epithelium of Muc4^−/−^ mice was intact, and the number of cell layers was the same as WT littermates (Fig. 1C). The corneal stroma of *Muc4*^−/−^ mice had a typical pattern of collagen lamellae with interspersed cells, similar to their WT littermates (Fig. 1C).

### Clinical staining and evaluation of smoothness

**Figure 2:**
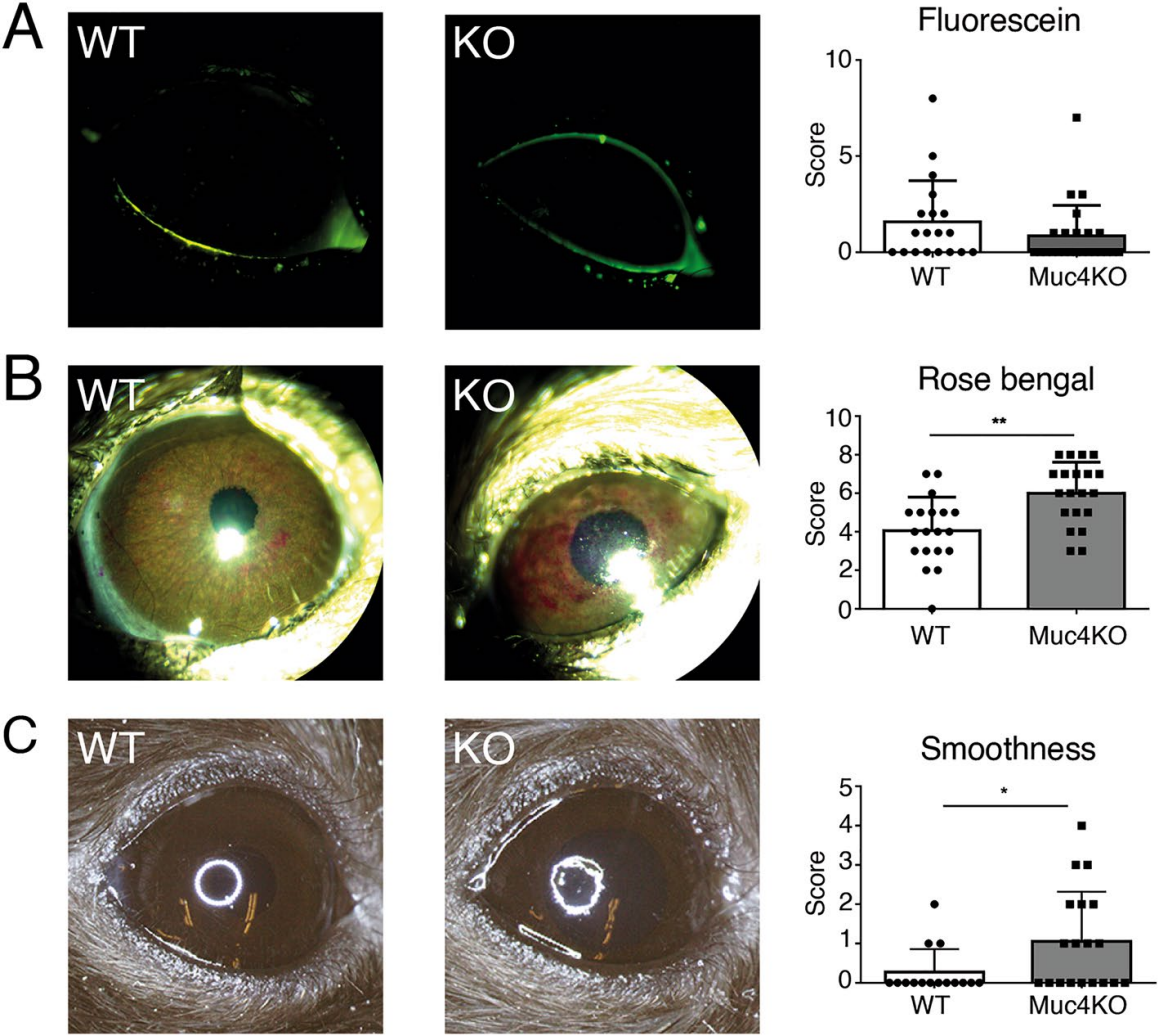
Macroscopic evaluation of *Muc4* KO mice. Representative images and quantification of (**A**) fluorescein staining, (**B**) rose bengal staining and (**C**) corneal smoothness of WT and *Muc4* KO mouse eyes. The data are presented as mean ± standard deviation. N = 20. *P < 0.05; **P < 0.01.

We next evaluated the ocular surface using non-invasive clinical tests. Representative results are shown in Fig. 2. First, we used fluorescein staining, which primarily measures superficial punctate keratopathy^38^, i.e., damage to individual epithelial cells and the tight junctions between them^39,40^. No significant difference in fluorescein staining was observed in *Muc4*^−/−^ mice as compared to WT littermates (Fig. 2A).

Next, we used rose bengal staining, which distinguishes the disruption of the mucosal glycocalyx in cultured cells^20^. In contrast to fluorescein staining, rose bengal staining was significantly elevated in *Muc4*^−/−^ mice, with a punctate pattern indicative of individual cells and cell cluster staining (Fig. 2B). Finally, we evaluated the smoothness of the corneal surface. This method has been used to evaluate tear film fluidity^41^. To this end, we examined the reflection of a ring of light on the cornea under a stereo microscope. Corneal smoothness was more frequently disrupted in *Muc4*^−/−^ mice corneas as compared to their WT littermates (Fig. 2C).

### Scanning electron microscopy and morphometric analysis

**Figure 3:**
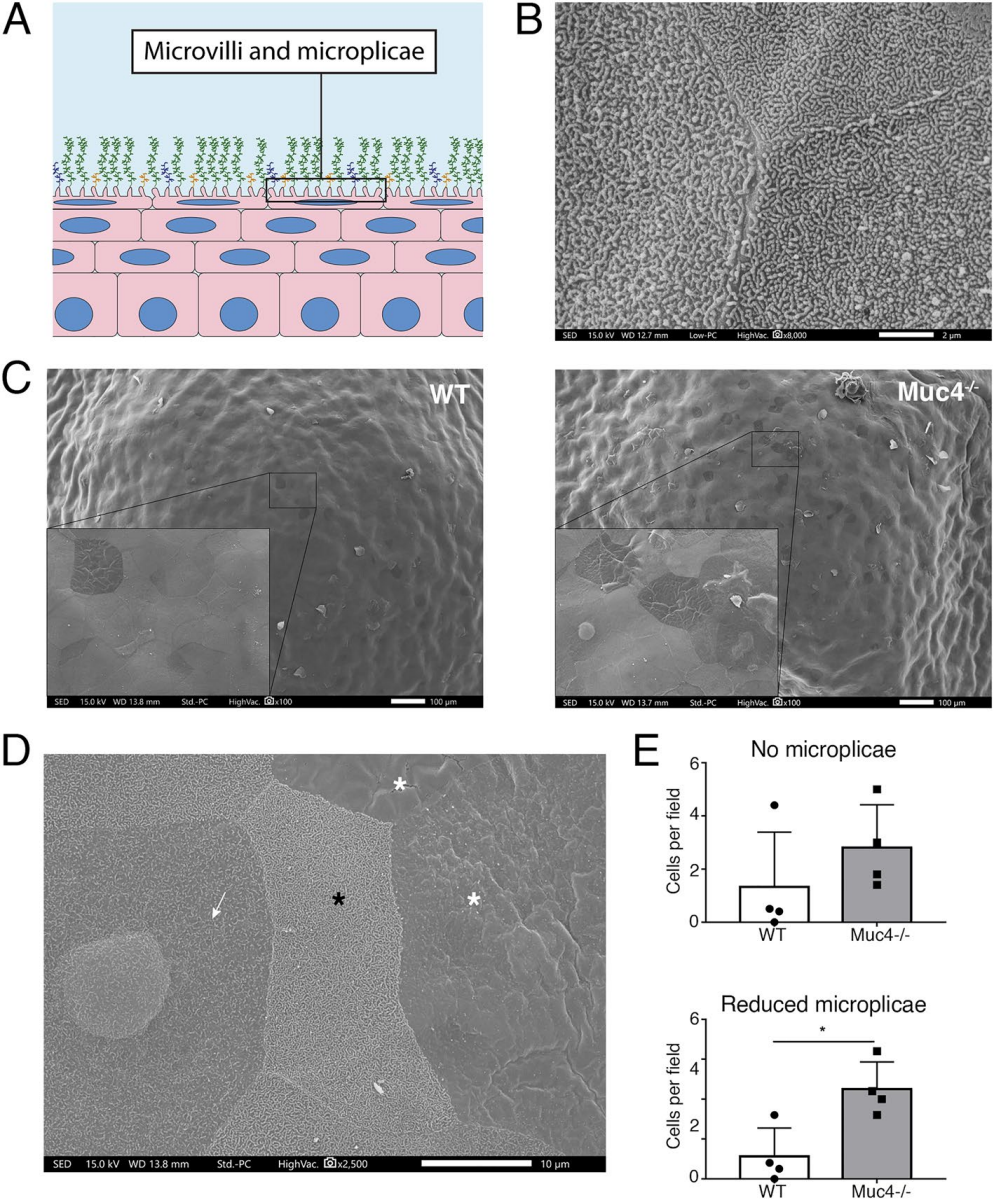
Ultrastructure analysis with scanning electron microscopy. (**A**) Schematic depicting the situation of membrane modifications in apical corneal epithelial cells. (**B**) Detail of the surface of three apical epithelial cells at high magnification, showing normally formed microplicae. This image was from a *Muc4* KO cornea, but WT corneas appeared identical. (**C**) Representative, low magnification images of the whole corneal surface in WT and *Muc4* KO mice. Detail of representative areas, evidencing the higher presence of darker cells in *Muc4* KO mice. (**D**) High magnification image of the *Muc4* KO mouse ocular surface showing the three different types of cells observed considering microplicae density: high microplicae density (black asterisk), reduced microplicae (arrow) and no microplicae (white asterisk). The ocular surface of WT mice looked similar. (**E**) Quantification of the number of “no microplicae” and “reduced microplicae” cells per field in WT and *Muc4* KO mice. The data are presented as mean ± standard deviation. N = 4; *P < 0.05.

We used scanning electron microscopy (SEM) to compare the surface architecture of *Muc4*^−/−^ and WT mouse eyes. Representative results are shown in Fig. 3. Much as in humans^21^, the apical cell layer of the mouse corneal epithelium presents plasma membrane microprojections^42^, as we show in the cross-sectional drawing (Fig. 3A). When viewed at high magnification, microprojections of normal appearance were found in both WT and *Muc4*^−/−^ mice, although with a variation in density apparent on individual cells (Fig. 3B). An image only from a *Muc4*^−/−^ mice is shown here, since WT mice looked identical. However, when the ocular surface was viewed at a lower magnification, it became apparent that there were more darker cells with lower microprojection density in *Muc4*^−/−^ mice (Fig. 3C). Examples can be found in Fig. 3D, with bright cells showing high density (Fig. 3D, black asterisk), grey cells showing reduced density (Fig. 3D, arrow) and dark cells being completely smooth (Fig. 3D, white asterisks).

We did a morphometric analysis to compare the number of bright, gray, and dark cells (smooth cells) per 1000 × field in *Muc4*^−/−^ and WT mice. While we observed a trend toward an increased number of smooth cells in the KO mice, this difference was not statistically significant. However, we found a significant increase in the number of cells with a low density of microplicae (gray cells) in *Muc4*^−/−^ corneas (Fig. 3E).

### Cornification, inflammatory, and transdifferentiation markers

**Figure 4:**
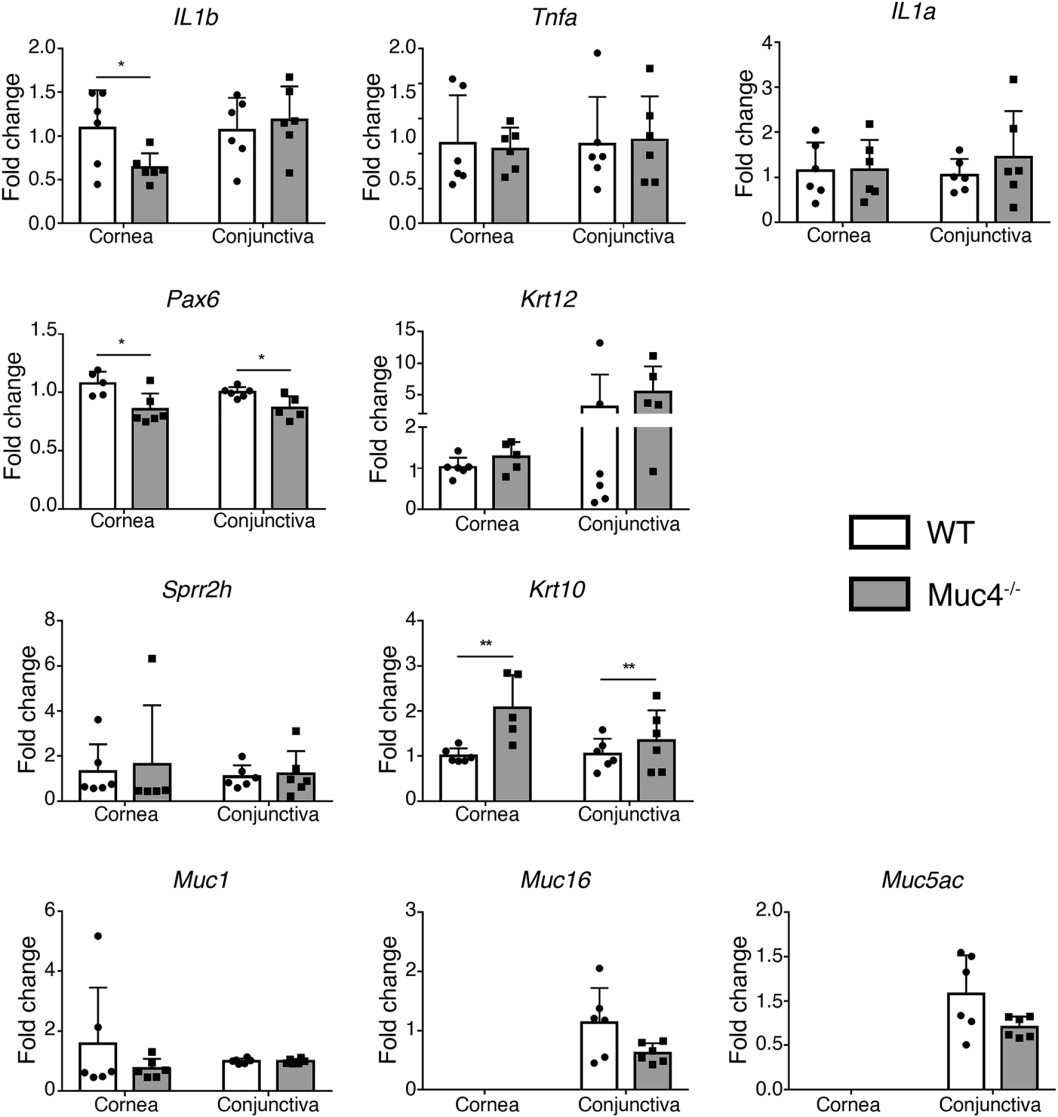
Gene expression analysis of the *Muc4* KO mouse ocular surface. Expression of mucin genes, epithelial differentiation markers and inflammatory markers in corneal epithelium and whole conjunctivas from WT and *Muc4* KO mice. Relative gene expression was calculated with the 2 − ΔΔCt method, using the levels of *Rpl9* expression as housekeeping and the expression in WT tissue as the calibrator. The data are presented as mean ± standard deviation. N = 6; *P < 0.05; **P < 0.01.

Dry eye disease is characterized by upregulation of pro-inflammatory cytokines and markers of cornification^41^. In severe dry eye disease subtypes like Stevens-Johnson syndrome, ocular cicatricial pemphigoid, and Sjögren’s syndrome, the wet mucosal epithelium can transdifferentiate to a keratinized epidermal-type phenotype^43,44^. We compared the expression of pro-inflammatory, cornification and epidermal transdifferentiation markers in Muc4^−/−^ mice to WT littermates by qPCR. Representative results are shown in Fig. 4. We observed no significant difference in the expression of genes encoding cytokines *Tnfa* and *Il1a* between the two groups. Expression of *Il1b* was significantly decreased in the cornea of *Muc4*^−/−^ mice as compared to WT littermates. Consistent with this, we observed no change in the expression of the cornified *Sprr2h*, and *Sprr1b* was undetectable in both *Muc4*^−/−^ and WT mice. Similarly, the cornea-specific keratin Krt12 expression was unchanged in *Muc4*^−/−^ mice compared to WT littermates. Interestingly however, expression of *Krt10*, an epidermal keratin, was significantly increased, while *Pax6*, a transcription factor that regulates corneal epithelial differentiation, was significantly decreased in both the cornea and conjunctiva of *Muc4*^−/−^ mice.

One of our labs previously showed that mice lacking *Muc4* upregulate other mucin genes in the colon epithelium when challenged with dextran sodium sulfate^35^. Since this can compensate for the effects of *Muc4* knockout, altering the phenotype, we investigated whether it also occurs at the ocular surface. However, qPCR analysis revealed no change in *Muc1* expression in the cornea or conjunctiva of *Muc4*^−/−^ mice compared to WT littermates (Fig. 4). Similarly, *Muc16* and *Muc5ac* expression levels were unchanged in the conjunctiva and remained undetectable in the cornea.

### Challenge with lipopolysaccaride (LPS)

**Figure 5:**
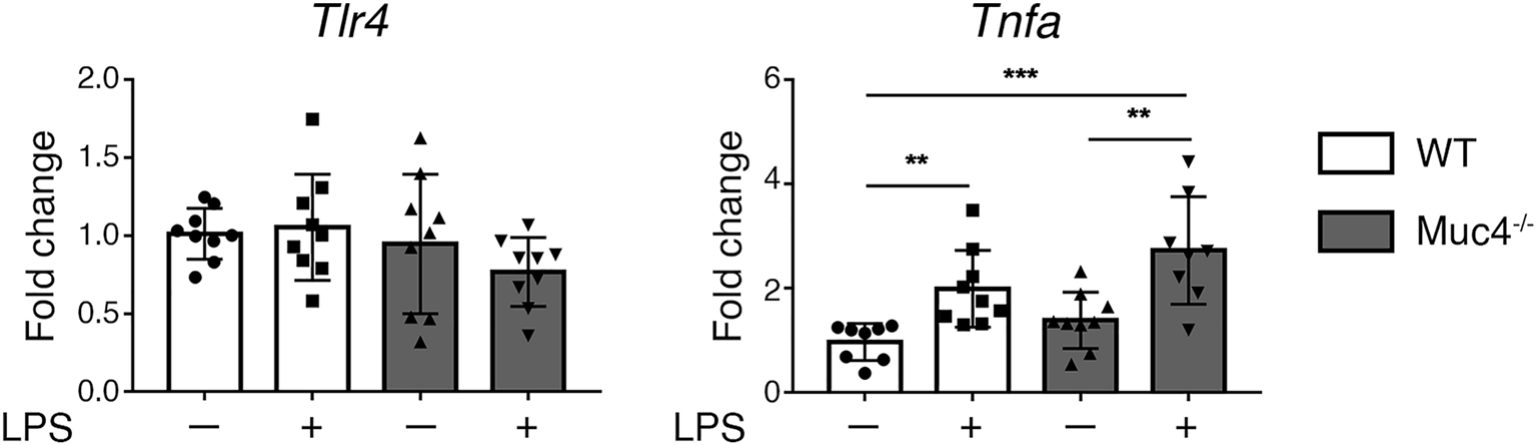
WT and *Muc4* KO eyes ex vivo exposure to LPS. WT and *Muc4* KO eyes were exposed to 1 μg/ml LPS for 4 h at 37 °C. Relative gene expression of *Tlr4* and *Tnfa* in the corneal epithelium was calculated with the 2 − ΔΔCt method, using the levels of *Rpl9* expression as housekeeping and the expression in untreated-WT tissue as the calibrator. The data are presented as mean ± standard deviation. N = 9; **P < 0.01; ***P < 0.001.

We tested the ability of Muc4 to interfere with TLR signaling by removing eyes from *Muc4*^−/−^ and WT mice to organ culture and exposing to LPS, an agonist of Tlr4. Representative results are shown in Fig. 5. No difference in Tlr4 expression was found in the corneal epithelial cells of unchallenged *Muc4*^−/−^ mice compared to WT littermates. Exposure to 1 µg/ml LPS for 4 h significantly increased the expression of *Tnfa* in the corneal epithelium in both *Muc4*^−/−^ and WT mice. There were no significant differences in the *Tnfa* expression increase between the LPS-treated *Muc4*^−/−^ and WT eyes.

## Discussion

Based on biophysical, cell/organ culture, and observational evidence, MAMs have been proposed to play critical roles at the ocular surface in tear film stability^7,11^, transcellular barrier function^20,21^, apical epithelial cell architecture^21^, dry eye pathology^14^ and dampening of the immune response^27^. Here we investigated these hypotheses by evaluating the phenotype of transgenic *Muc4* KO mice. Loss of Muc4 at the ocular surface compromised transcellular barrier function, and we also found evidence of tear film disruption. In addition, loss of Muc4 altered the architecture of the apical epithelial cell layer, as evidenced by an increase in cells with fewer microplicae. We also found evidence of an incipient transdifferentiation of the corneal epithelium to an epidermal phenotype. These results provide the first in vivo evidence supporting several of the long-standing hypotheses cited above. However, our findings did not support observational studies linking loss of MAMs to dry eye pathology. Moreover, the loss of Muc4 did not dampen the immune response mediated by tlr5.

The targeting approach for the *Muc4* KO mice used in this study employed a knock-in strategy by insertion of a bacterial beta-galactosidase (LacZ) transgene in the endogenous *Muc4* locus, placing it under the control of the *Muc4* promoter^35^. Histochemical analysis of *Muc4* promoter activity revealed a spiraling ribbon pattern, consistent with the known centripetal migration of epithelial cells to the central cornea from the limbus. This expression pattern is similar to that shown for genes that regulate cell fate in the cornea (e.g.,^45^). While in situ hybridization has been used to visualize MAMs expression at the ocular surface, and results of this study are consistent with previous findings for *Muc4* expression^33,36^, this is the first time to our knowledge, that MAM gene expression has been visualized in a 2D coronal view, providing new information.

It has been hypothesized that MAMs with very long EDs have specialized roles that MAMs do not serve with short EDs^22^. The clustering of O-linked oligosaccharide chains within their tandem repeats creates steric interactions between carbohydrates and peptides, inducing the peptide core to adopt a stiff and extended conformation. This results in projection well above the cell surface, far beyond other membrane-associated proteins^46^. The extracellular domain of human MUC4 is predicted to extend > 2 um above the cell surface in the apical region^47^. Thus, MAMs with very long EDs are positioned to shield and protect the cell surface and also create a transcellular barrier^20,21^.

MUC4/Muc4 and MUC16/Muc16 are the MAMs with very long EDs expressed at the ocular surface of humans and mice^17,18^. In humans, *MUC16* mRNA has been reported to be expressed evenly across the corneal and conjunctival epithelia^48,49^. However, in mice, *Muc16* expression has been identified only in the conjunctival epithelium^34^. *MUC4* mRNA is most abundant in the human conjunctiva, with an attenuation in expression from the corneal periphery to the central corneal^50,51^. Muc4 is expressed in both mouse conjunctiva and cornea^36^. Thus, Muc4 appears to substitute for MUC16 in the mouse corneal epithelium.

MAM transcellular barrier function is thought to be dependent on a very long and heavily glycosylated ED, which excludes small molecules^20^. Knockdown of MUC16 in a human cell culture model resulted in transcellular barrier disruption, as evidenced by increased rose bengal penetrance^20,21^. In contrast, knockdown of MUC1, a short ED MAM, decreased rose bengal penetrance, perhaps because its interspersion with MUC16 creates spaces in the barrier^21^. Our finding of transcellular barrier disruption in *Muc4* KO mice provides the first in vivo support for this MAM role.

While Muc4 may substitute for Muc16 for some functions, this may not always be the case. Thus while both MUC4/Muc4 and MUC16/Muc16 share the feature of very long EDs, and both have cleavage sites for shedding into the tear film, the overall modular structures of their EDs are quite different^17^. For example, the ED of MUC4/Muc4 has three EGF-like motifs located distal to the cleavage site, which is not present in MUC16/Muc16^52^. Rat Muc4 was shown to interact with EGFR family member ERBB2 via the EGF-like motif closest to its transmembrane domain, resulting in phosphorylation and downstream signaling^53^. The EGF-like motifs are not found in the MUC16/Muc16 ED. Similarly, while both CTs are short, the few identified motifs affecting intracellular signaling differ in the CTs of the two MAMs^17^. Thus, Muc4 substitution for MUC16 in the corneal epithelium of mice may have functional significance in some cases.

Scanning electron microscopy of the mammalian corneal surface has revealed a contiguous mosaic of polygonal cell shapes with a range of sizes, each having a light, medium, or dark appearance^42,54,55^. Lighter cells have a greater density of microprojections, to which MAMs localize, while the darkest cells are entirely smooth. These differences are thought to reflect cell maturation that starts when a cell reaches the ocular surface and ends when it is desquamated^42,54,55^. It has been proposed that cells with more microprojections are younger cells, which gradually flatten as they mature^36,51,52^. When viewed by transmission electron microscopy (TEM), the shades are reversed, with the cytoplasm of light cells being electron dense, consistent with a greater metabolic and synthetic activity, while dark cells appear to have reduced metabolic activity, consistent with the idea that they are more mature^42^. Here, we observed the typical pattern of light, medium and dark cells at the corneal surface of both WT and Muc4 KO mice. As in the *Muc16* KO mouse, well-developed microprojections were apparent in both genotypes^34^. However, morphometric analysis revealed that loss of Muc4 results in more cells with reduced microprojection density. The shift to more cells with reduced microprojection density was not observed in the *Muc16* KO mouse^34^, but since Muc16 is primarily localized to the conjunctival epithelium in mice^17^, an effect on the microprojections would be precluded. Interestingly, knockdown of MUC16 in a human corneal epithelial cell culture model resulted larger, more spread cells with reduced cell surface microprojections^21^. Pull-down experiments suggested that a polybasic amino acid stretch at the proximal end of the MUC16 CT interacts with ezrin/radixin/moesin (ERM) family actin-binding proteins^20^, a family known to contribute to the formation of microprojections^56,57^. However, MUC4/Muc4 CT lacks the ERM actin-binding motif^17^. Moreover, our observation was not a loss of microprojections overall, but a shift towards more cells with reduced microprojections, a somewhat different effect that suggests a different mechanism.

It seems possible that the shift towards more cells with reduced microprojections could be related to shear stress. Corneal epithelial cells are constantly exposed to shear stress due to blinking. The apical surface of differentiated human corneal epithelial cells expressing *MUC16* was shown to be more antiadhesive than undifferentiated cells lacking MUC16 and abrogation of mucin O-glycosylation in differentiated cultures resulted in increased adhesion^58^. Thus a reduction in Muc4 could reduce the lubrication and increase the shear stress caused by normal blinking. Under flow-induced shear stress, cells were larger and more spread as compared to static monolayer controls^59^. MAMs and the microprojections to which they localize are thought to help stabilize the tear film^1^. Thus the loss of Muc4 and its effect on the overall density of microprojections across the ocular surface is consistent with our observation of reduced tear film stability in *Muc4* KO mice.

While expression of the corneal epithelial keratin marker K12 was unchanged in this study, we found an increase in the epidermal-type keratin K10 and a decrease in the eye-specific transcription factor Pax6. This apparent incipient keratinization in mice lacking Muc4 may be caused by the resulting reduced tear film stability and increased shear stress. K10 increase is one of the first steps in epidermal cornification: the keratins K1 and K10 form scaffolds where the cornifins will bind to form the cornified envelope^60^. Keratinization and cornification are hallmarks of squamous metaplasia that occurs at the ocular surface due to the desiccation and inflammation of dry eye^43,61,62^. Numerous observational studies have reported that MAMs are quantitatively or qualitatively deficient in dry eye disease^14–16^ and severe dry eye can result in transdifferentiation of mucosal epithelial cells to a skin phenotype^63^. Interestingly, we observed no other hallmarks of dry eye, including epithelial cell damage as visualized by fluorescein staining, goblet cell loss, or increased expression of inflammatory cytokine and cornification markers. This suggests the intriguing hypothesis that Muc4 is required to maintain mucosal epithelial differentiation, over and above any role in inflammatory diseases such as dry eye.

Not only did we observe no increase in expression of genes encoding inflammatory cytokines in *Muc4* KO mice as compared to WT littermates, expression of the gene encoding the inflammatory cytokine *Il1b* was reduced. This is consistent with previous findings from one of our labs using the dextran sodium sulfate (DSS)-induced colitis model, in which *Muc4* KO mice displayed reduced infiltration of inflammatory cells along with a reduction in mRNA encoding inflammatory cytokines in the inflamed colon mucosa as compared with WT littermates^35^. Compensatory upregulation of *Muc2* and *Muc3* under basal and DSS treatment conditions partly factored into this phenotype. Significantly, we did not observe compensatory upregulation of secreted mucin genes or MAMs at the ocular surface in the current study. Increased inflammation was reported at the ocular surface of the *Muc16* KO mice, however, as in this study, no other signs of dry eye disease were observed^34^.

A limitation of the current study is that we examined only very young mice. It is intriguing also that inflammation was not seen in the KO mouse at baseline given that there appear to be epithelial defects. Perhaps dry eye disease might take more time to develop in MAM KO mice and examination of older mice might reveal disease signs. Testing these mice in a desiccating environment as well as their response to corneal debridement or wounding might also reveal fundamental roles for Muc4 in corneal health. Additional markers of keratinization and dry eye, evaluated not only by qPCR markers, but also by immunoblotting, ELISA and immunohistochemistry would be needed to thoroughly test the mucosal maintenance hypothesis.

MUC1/Muc1 and MUC16/Muc16 are known to inhibit the TLR response to challenge^27,64^. However, we found no difference in response to challenge with the Tlr4 agonist LPS in *Muc4* KO mice or WT littermates. The differential contributions of the different MAMs to TLR activity and general inflammation suggest that changes in the proportions of these mucins can lead to very different responses to noxious stimuli and even allergens, some known to activate TLRs in ocular epithelial cells.

In conclusion, the results of this study provide the first in vivo evidence for several proposed MAM roles at the ocular surface. First, it is demonstrated that loss of Muc4 compromises transcellular barrier function. Determining the basic mechanisms that create and sustain the mucin transcellular barrier is relevant not only for addressing the negative clinical consequences of its alteration but also for improving drug delivery, as mucins are a significant impediment to the delivery of topical drugs in the eye^65,66^. Second, our results support the findings of biophysical studies on the requirement of MAMs for tear film stability. Third, we report effects of Muc4 loss on apical epithelial cell architecture, which may be due to the anti-adhesive role of MAMs, previously proposed based on in vitro evidence. Fourth, we obtain evidence that loss of Muc4 results in incipient keratinization, suggesting the hypothesis that Muc4 is needed to maintain mucosal differentiation at the ocular surface. Surprisingly, we found no evidence that this is accompanied by other signs of dry eye, challenging the generally accepted paradigm. Follow-up work is necessary to fully test the tentative conclusions of this fourth set of findings. Finally, we show that not every MAM can suppress the immune response through toll-like receptors.

## Materials and methods

### Animals

All animal experiments conformed to the ARVO Statement for the Use of Animals in Ophthalmic and Vision Research and to the recommendations of the National Institutes of Health Guide for the Care and Use of Laboratory Animals. The study was in compliance with ARRIVE guidelines. The breeding and animal procedures were approved by the IACUC of Tufts University.

This study made use of *Muc4* KO mice, previously generated by targeted disruption in the Batra laboratory at the University of Nebraska Medical Center (Omaha, NE)^35^. Homozygous *Muc4*^−/−^ mice were shown to be viable and fertile, with no apparent defects. qPCR using primers to the 3ʹ region of the transcript confirmed the lack of *Muc4* expression in normal colon and lungs of *Muc4*^−/−^ mice, comparing to *Muc4*^+/+^ mice that express this gene (positive control), and the lack of pancreatic expression in both *Muc4*^−/−^ and *Muc4*^+/+^ mice (negative control).

Heterozygous *Muc4*^+/−^ males on the C57Bl/6 background were imported to Tufts Medical Center (Boston, MA), then back-crossed with C57Bl/6 females to generate sufficient heterozygotes to expand the colony. Heterozygotes were then crossed to generate sufficient homozygous KO mice (*Muc4*^−/−^) and WT littermates (*Muc4*^+/+^) for experiments. Mice were housed on a 12-h light–dark cycle with food and water available ad libitum. Eight- to twelve-week-old mice, with an approximately equal male/female proportion, were used for all experiments.

### Histochemistry

For histochemical localization of beta-galactosidase activity, a kit was utilized following the manufacturer's instructions (Abcam, Cambridge, UK). The whole eyes were carefully collected and immediately placed in Fixative Solution for one hour at room temperature. Then, the corneas were gently separated from the rest of the eye and washed twice in PBS before being incubated in freshly prepared Staining Solution overnight at 37 °C. After the overnight incubation, the corneas were thoroughly washed in PBS, mounted, and observed under a microscope.

### Histology

Wild type and homozygous *Muc4* KO mice (5 per group) were euthanized and the eyes with eyelids were collected and fixed in 4% paraformaldehyde for 4 h at room temperature. The fixed eyes were then cryoprotected in 30% sucrose and frozen in OCT®. Sections of 10 μm thickness were obtained using a cryostat. Hematoxylin & eosin staining (Vector) was performed on the sections and evaluated under a microscope.

### Clinical staining

For the staining of the ocular surface, mice were anesthetized using an intraperitoneal injection of ketamine (100 mg/kg) and xylazine (10 mg/kg). The eyes were then observed and photographed using a Phoenix Micron IV with a slit-lamp attachment (Phoenix Research Laboratories, Pleasanton, CA) using white and cobalt blue light as needed. For fluorescein staining, a single drop of 0.35% fluorescein was carefully instilled onto the ocular surface and, after 2 min, the excess of dye was washed with phosphate-buffered saline (PBS). Similarly, for rose bengal staining, one drop of 1% rose bengal was applied and allowed to stand for 30 s before being washed away with PBS. A modified van Bijserveld scoring system was used to assess the degree of staining. The total score went from 0 to 9, with the cornea divided in 3 areas, and each area scored from 0 to 3, with 0 indicating no staining, 1 indicating sparsely scattered staining, 2 indicating densely scattered or scattered plaques, and 3 indicating confluent or diffuse staining or diffuse plaques.

### Evaluation of corneal smoothness

To evaluate the smoothness of the eyes, we used a previously described method that involved examining the reflection of a white ring under stereomicroscopy^41^. The eyes were imaged immediately after euthanasia to minimize the effects of post-mortem changes. The reflection of the ring was scored on a scale of 0 to 5, with a score of 0 indicating no distortion and a score of 5 indicating complete distortion or loss of the ring reflection. Scores of 1, 2, 3, and 4 represented increasing levels of distortion in one quarter, half, three-quarters, and all quarters of the ring, respectively. Smoothness was scored considering the alteration of a ring light reflection, as has been described before^41^. Immediately after euthanasia, images of the eyes reflecting the light of a white ring of a stereomicroscope were taken. The images were scored from 0 to 5, with the scoring meaning (0) no distortion of the ring, (1) distortion in one quarter of the ring, (2) distortion in half of the ring, (3) distortion in three quarters, (4) distortion in the four quarters and (5) when no ring could be recognized.

### Scanning electron microscopy

Eyes from four *Muc4*^−/−^ mice and four *Muc4*^+/+^ littermates were collected and fixed in 1/2 strength Karnovsky fixative, dehydrated through ethanol series, and then subjected to critical point drying with a SamDri-795 critical point dryer (Tousimis Research Corporation, Rockville, MD). The dried eyes were coated with chromium using an Ion Beam Coater 610 (Gatan Corp. Pleasanton, CA). Consequently, eyes were observed and photographed under a scanning electron microscope. For analysis, five different fields were randomly selected from each cornea and photographed at 1000 × magnification. The number of cells per field with no microplicae or reduced microplicae were counted.

### RNA isolation and quantitative polymerase chain reaction (qPCR)

The conjunctiva was collected from both eyes using forceps and micro-scissors. The cornea was removed to collect the epithelium by scrapping it with a blade after incubating it in 20 mM EDTA for 10 min.

RNA was isolated using the GeneJET RNA Purification Kit (Thermo Fisher Scientific) following the manufacturer's instructions. The corneal epithelia were homogenized by 15 s of vortexing, while conjunctivas were passed through a syringe attached to a 20-G needle. To remove DNA contamination, PureLink® DNase Set (Invitrogen, Carlsbad, CA) was used on columns. First-strand cDNA was synthesized using the High Capacity Reverse Transcription Kit (Applied Biosystems, Foster City, CA), following the manufacturer's instructions.

The RT-qPCR reaction was carried out using SYBR® Green reagent (iTaq Universal SYBR Green Supermix, Bio-Rad, Hercules, CA) with specific primers (Table 1). The following parameters were used: 30 s at 95 °C, followed by 40 cycles of 5 s at 95 °C and 30 s at 60 °C. All samples were normalized to RNA levels of *Rpl9* gene as the housekeeping (Table 1). The comparative CT method was used for relative quantitation, selecting the relative amount in WT mice as the calibrator.

**Table 1:** Primer sequences for RT-PCR.

Gene	Primer sequence
*Muc1*	Fwd: CCTACCATCCTATGAGTGAATACC Rev: GACTGCTACTGCCATTACCTG
*Muc16*	Fwd: AAGTTCAAAACCCACTGGGGA Rev: ATGGGTTTGTAGTTGGCCTT
*Muc5ac*	Fwd: CCCATGTGTATTCCTCTCCCA Rev: CTGGTTGAGTGGTTGTGTGT
*Pax6*	Fwd: AGTGAATGGGCGGAGTTATG Rev: ACTTGGACGGGAACTGACAC
*Krt12*	Fwd: GTAAATACTACCCACTGATTGAAGAC Rev: GCCAGCTCATTCTCATACTTCA
*Krt10*	Fwd: CTACAAAACCATCGAGGACCT Rev: CCTCATTCTCGTATTTCAGCCT
*Sprr2h*	Fwd: CAAGCTCTGGACTAAGGAGAAC Rev: TGGGCACACAGGAGGAG
*Il1b*	Fwd: CAACCAACAAGTGATATTCTCCAT Rev: GGGTGTGCCGTCTTTCATTA
*Tnfa*	Fwd: AAGCCTGTAGCCCACGTCGTA Rev: GGCACCACTAGTTGGTTGTCTTTG
*Il1a*	Fwd: CTGCAGTCCATAACCCATGA Rev: ACAAACTTCTGCCTGACGAG
*Rpl19*	Fwd: ATGCCAACTCCCGTCAGCAG Rev: TCATCCTTCTCATCCAGGTCACC

### LPS exposure

Eyes of *Muc4*^+/+^ and *Muc4*^−/−^ mice were enucleated and incubated in keratinocyte serum-free medium (Gibco-Thermo Fisher Scientific, Waltham, Massachusetts, USA) containing 1 μg/ml LPS (Sigma-Aldrich, St Louis, MO, USA) for 4 h at 37 °C. The corneal epithelia were then collected as explained above and RNA was extracted. The relative gene expression of *Tlr4* and *Tnfa* in the corneal epithelium was calculated by RT-qPCR, using *Rpl9* as the housekeeping gene for comparison and WT-untreated eyes as the calibrator.

### Statistical analysis

Statistical analysis was performed using GraphPad Prism 7 (GraphPad Software, San Diego, CA, USA). Student t-test or Mann–Whitney *U* test were used attending to normality of the data dis-tribution as determined by using the D’Agostino & Pearson normality test. Analysis of Variance (ANOVA) with Bonferroni's post-hoc test was applied for comparison of multiple samples. P value < 0.05 was considered statistically significant.

## Data Availability

All data generated or analyzed during this study are included in this published article.
